# Reduced Circulating Levels of miR-491-5p and miR-485-3p Are Associated with the Occurrence of Vertebral Fractures in Postmenopausal Women with Osteoporosis

**DOI:** 10.1155/2022/3838126

**Published:** 2022-03-07

**Authors:** Jixi Xu, Mingbo Li, Wei Pei, Jinyong Ding, Yueran Pan, Huifeng Peng, Shiman Lin, Yanbo Huang

**Affiliations:** ^1^The First Institute of Clinical Medicine, Guangzhou University of Chinese Medicine, Department of Spinal Surgery, The First Affiliated Hospital of Guangzhou University of Chinese Medicine, Guangzhou, China; ^2^The First Institute of Clinical Medicine, Guangzhou University of Chinese Medicine, Guangzhou, China; ^3^ShunDe Hospital GuangZhou University of Chinese Medicine, Foshan, China; ^4^Huizhou Occupational Disease Prevention and Treatment Institute, Huizhou, China; ^5^Hospital of Stomatology, SunYat-sen University, Guangzhou, China

## Abstract

**Objective:**

Postmenopausal women experiences osteoporotic structural damage and bone fragility resulting from reduced bone formation and increased bone resorption. Osteoporosis frequently affects the vertebral column and causes compression fractures. This study aims to characterize roles of miRNAs in osteoporosis and subsequent incidence risk of vertebral fractures for postmenopausal women. Methods. Differentially expressed miRNAs between osteoporotic patients with vertebral fractures and osteoporotic patients without fracture were identified. This retrospective study included 78 osteoporotic patients with vertebral fractures and 82 osteoporotic patients without vertebral fractures. The plasma levels of bone metabolic markers, 25-hydroxyvitamin D (25-(OH)VitD), propeptide of type I procollagen (PINP), and β-Carboxyl terminal peptide (β-CTx), were detected using the patented electro-chemiluminescence (ECLIA) method. The expression levels of miR-491-5p and miR-485-3p were determined by qRT-PCR. Pearson correlation analysis was carried out to assess the relationship between miR-491-5p, miR-485-3p, and bone metabolic markers. Receiver operating characteristic (ROC) curves and the area under the ROC curve (AUC) were used to evaluate the performance of miR-491-5p and miR-485-3p in diagnosing the occurrence of vertebral fractures in osteoporotic patients.Results: The plasma levels of PINP and β-CTx were elevated but the plasma level of 25-(OH)VitD was declined in osteoporotic patients with vertebral fractures when comparable to those without (< 0.05). The plasma expression levels of miR-491-5p and miR-485-3p were declined osteoporotic patients with vertebral fractures when comparable to those without (< 0.001). Pearson correlation analysis revealed that the relative expression level of miR-491-5p was negatively correlated with the level of 25-(OH)VitD (r = -0.518, < 0.001) but positively correlated with the levels of PINP (r = 0.547, < 0.001) and β-CTx (r = 0.380, < 0.001). We also observed a negative correlation between the relative expression level of miR-485-3p and 25-(OH)VitD (r = -0.388, < 0.001), a positive correlation between miR-485-3p and PINP (r = 0.422,< 0.001). ROC curves for prediction of vertebral fracture following osteoporosis in postmenopausal women by miR-491-5p expression yielded 0.866 AUC and by miR-485-3p expression produced 0.848 AUC.

**Conclusion:**

The data suggest that downregulated expressions of miR-491-5p and miR-485-3p may be involved in the occurrence of vertebral fractures in postmenopausal women with osteoporosis.

## 1. Introduction

Osteoporosis is a progressive systemic disease that is associated with bone mass decline and degradation of the bone microarchitecture, resulting in enhanced bone fragility and a greater fracture risk [1]. The incidence rate of osteoporosis increases with aging, and 30% of postmenopausal women are affected by osteoporosis in their remaining lifetime from the age of 50 years largely resulting from hormone estrogen deficiency after menopause [2]. Several factors have been known to be associated with an increasing risk of fracture, especially of the spine and hip, in postmenopausal women with osteoporosis, including the history of fracture, advancing age, decreased bone mineral density (BMD), higher risk or history of falls, and certain pharmacologic therapies, such as glucocorticoids [3, 4]. Up to 40% of women with an increased risk of fracture take antiosteoporosis drugs according to the Global Longitudinal Study of Osteoporosis in Women [5]. In addition to sufficient exercise to enhance muscle function, women should ensure adequate calcium intake and vitamin D [1]. Menopausal hormone therapy significantly prevents osteoporosis and the consequent fractures and should be encouraged in those aged less than 50 years. With regard to women aged less than 60 years, MHT or tibolone can be suggested, especially the manifestations of vasomotor or genitourinary symptoms. Concerning those aged over 60 years, risedronate or bisphosphonates may then be considered [6]. The diagnostic tools currently available for fragility fractures, such as dual energy X-ray absorptiometry, fracture risk assessment tool (FRAX) score, and bone turnover markers including bone alkaline phosphatase (ALP), procollagen type I N-terminal propeptide (PINP), and serum total osteocalcin, have limited specificity and sensitivity, which indicates the requirement to develop alternative approaches [7]. Circulating cell-free microRNAs (miRNAs) are promising new potential biomarkers for diagnosing osteoporosis and assessing low-traumatic fracture risk due to advantageous features, such as noninvasiveness, biofluid stability, and easy detection [8].

MicroRNAs (miRNAs) represent a class of epigenetic regulators that posttranscriptionally modulate and silence target gene expression, thus regulating a variety of biological events essential for bone homeostasis, such as osteoclast differentiation and osteoblast-to-osteoclast communication [9, 10]. In recent years, accumulating evidence has demonstrated the presence of stable cell-free mature miRNAs in blood and shows that the alternations in their serum/plasma levels can indicate both physiological and pathological states, thus making them useful candidate molecules as noninvasive biomarkers in osteoporosis and bone fracture risk [11]. An aberrant c-miRNA expression profile has been associated with an altered BMD status and the presence of bone fractures [12]. Additionally, circulating miRNAs have been found to correlate with histomorphometric indices in patients with osteoporosis, thus possibly reflecting changes in bone microstructure [13]. Interestingly, tissues expression of several miRNAs is highly related with their serum expressions in osteoporotic patients, suggesting that expression of miRNAs may be indirectly associated with the bone metabolism [14, 15]. In this study, we first performed microarray dataset analysis to identify differentially expressed miRNAs in plasma between osteoporotic patients with vertebral fractures and osteoporotic patients without fracture. To characterize roles of candidate miRNAs in osteoporosis and subsequent incidence risk of vertebral fractures for postmenopausal women, we chose two differentially expressed miRNAs between osteoporotic patients with vertebral fractures and osteoporotic patients without fracture, miR-491-5p and miR-485-3p, and determined their expression levels in the plasma of osteoporotic patients with vertebral fractures and osteoporotic patients without vertebral fractures.

## 2. Materials and Methods

### 2.1. Microarray Dataset Analysis

A microarray dataset (accessioned as GSE93883) containing miRNAs profiles in osteoporotic patients with and without vertebral fractures was downloaded from the Gene Expression Omnibus, which was generated on the GPL18058 platform. The GSE93883 dataset includes a pool of 6 miRNA samples from osteoporotic patients with vertebral fractures and a pool of 6 miRNA samples from osteoporotic patients without fracture, aiming to identify differentially expressed miRNAs in plasma. Differentially expressed miRNAs that were upregulated or downregulated |log2 (fold change)|  ≥ 1 (corrected *P* < 0.05) between osteoporotic patients with vertebral fractures and osteoporotic patients without fracture were screened by analyzing raw data of GSE93883 using the affy and limma package from the R/Bioconductor software.

### 2.2. Study Participants

This retrospective study included 78 osteoporotic patients with vertebral fractures and 82 osteoporotic patients without vertebral fractures who were admitted into the First Affiliated Hospital of Guangzhou University of Chinese Medicine between January 2019 and June 2021. The diagnosis of postmenopausal osteoporosis was confirmed according to the guidance provided by the World Health Organization (WHO) [16]. According to BMD testing, the WHO clinically defines women with osteopenia as those whose BMD T-score is between 1 and 2.5 standard deviations (SD) below the mean of peak bone mass in healthy, young normal women (i.e., −1 to −2.5 SD); women with osteoporosis include all those with T-score ≤ −2.5 SD. The inclusion criteria were natural menopause, new onset vertebral fractures, and primary osteoporosis without bone metabolism-associated diseases. The exclusion criteria were long-term use of drugs affecting the bone metabolism, such as parathyroid hormone, calcitonin, hormone, the presence of serious diseases of the liver, kidney, heart, and cerebrovascular systems, abnormal immune function, malignancies, pregnancy, and mental illness. All included patients signed consent document prior to study inclusion. The study was approved and reviewed by the Ethics Committee of the First Affiliated Hospital of Guangzhou University of Chinese Medicine.

### 2.3. Measurements of Bone Mineral Density (BMD) and Bone Metabolism

Fasting venous blood (5 ml) was collected from each patient. After centrifugation (3000 r/min, 5 min), plasma was extracted. The lumbar and hip BMD was detected by DPX-MD dual energy X-ray bone densitometry (Luna, USA). The bone metabolism was evaluated by detecting the plasma levels of bone metabolic markers, 25⁃hydroxyvitamin D (25-(OH)VitD), propeptide of type I procollagen (PINP), and *β*⁃Carboxyl terminal peptide (*β*-CTx). The plasma levels of 25-(OH)VitD, PINP, and *β*-CTx were detected using the patented electrochemiluminescence (ECLIA) method by Cobas (Roche Diagnostics International Ltd., Basel, Switzerland) with a detection limit of 3 ng/ml on the Roche Elecsys 2010 Immunoassay Analyzer (Roche Diagnostics Ltd., Basel, Switzerland).

### 2.4. RNA Extraction and Quantitative Real-Time Polymerase Chain Reaction (qRT-PCR)

Total RNA was extracted from patient plasma sample using TRIzol reagent (Invitrogen, USA) following the supplier's instructions. The NanoDrop 2000 (Invitrogen) was employed to measure the purity and concentration of total RNA. The cDNA was generated by reverse transcription using the PrimeScript RT reagent kit (Takara, Japan). qRT-PCR was carried out using a SYBR Green I Master Mix kit (Invitrogen). All reactions were done in triplicate using the ABI 7300 machine (Applied Biosystems, USA) with the following thermocycling conditions: initial denaturation at 95°C for 10 min; 40 cycles of 95°C for 30 s, 60°C for 15 s, and 72°C for 15 s; a final extension at 72°C for 10 min, and the following primer sequences: miR-491-5p forward, 5′-CGAGTGGGGAACCCTTCC-3′ and reverse, 5′-AGTGCAGGGTCCGAGGTATT-3'; miR-485-3p forward, 5′-GCCGAGGTCATACACGGCTCTCCTCT-3′ and reverse, 5′-TGTCGTGGAGTCGGCAATTC-3'; U6 forward, 5′-CTCGCTTCGGCAGCACA-3′ and reverse, 5′-AACGCTTCACGAATTTGCGT-3′. Relative miR-491-5p and miR-485-3p expressions were observed using the 2^−ΔΔC^ method, with U6 used for normalization.

### 2.5. Statistical Analysis

Statistical comparisons of measurement data (mean ± standard deviation) were performed with SPSS 21.0 software (IBM, Armonk, NY, USA) using the unpaired *t*-test. Pearson correlation analysis was carried out to assess the relationship between miR-491-5p, miR-485-3p, and bone metabolic markers. Receiver operating characteristic (ROC) curves and the area under the ROC curve (AUC) were used to evaluate the performance of miR-491-5p and miR-485-3p in diagnosing the occurrence of vertebral fractures in osteoporotic patients.

## 3. Results

### 3.1. Differentially Expressed miRNAs between Osteoporotic Patients with and without Vertebral Fractures

**Figure 1:**
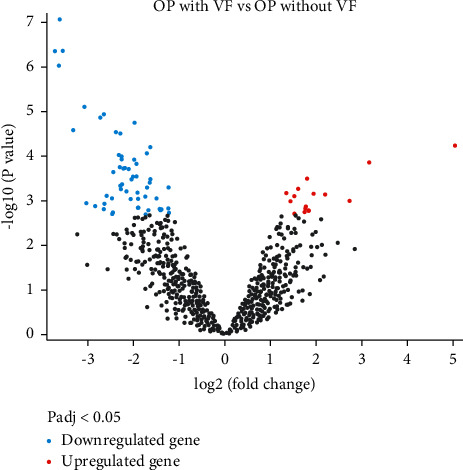
The volcano plot presenting differentially expressed miRNAs (|log2 (fold change)|  ≥ 2, adjusted *P* < 0.05) between osteoporotic patients with vertebral fractures and osteoporotic patients without fracture.

After differentially analysis, there were 12 miRNAs being upregulated and 25 miRNAs being downregulated |log2 (fold change)|  ≥ 1 (corrected *P* < 0.05) between osteoporotic patients with vertebral fractures and osteoporotic patients without fracture (Figure 1).  Table 1 provides differentially expressed miRNAs that were upregulated or downregulated |log2 (fold change)|  ≥ 2 (corrected *P* < 0.05) between osteoporotic patients with vertebral fractures and osteoporotic patients without fracture.

### 3.2. Patient Characteristics

The osteoporotic patients with vertebral fractures aged from 54 to 83 years, with an average age of (67.90 ± 7.04) years, and their age of menopause was (52.67 ± 2.78) years. The osteoporotic patients without vertebral fractures aged from 53 to 82 years, with an average age of (66.84 ± 6.58) years, and their age of menopause was (52.40 ± 2.76) years. The BMI, lumbar, and hip BMD of osteoporotic patients with vertebral fractures were 22.36 ± 2.94, 0.58 ± 0.05 (g/cm^2^), and 0.68 ± 0.05 (g/cm^2^). The BMI, lumbar, and hip BMD of osteoporotic patients without vertebral fractures were 22.39 ± 2.89, 0.5 ± 0.04 (g/cm^2^), and 0.67 ± 0.06 (g/cm^2^). Two groups of osteoporotic patients showed no significant difference in age, BMI, lumbar, and hip BMD.

### 3.3. The Plasma Levels of 25-(OH)VitD, PINP, and *β*-CTx in Osteoporotic Patients with or without Vertebral Fractures

In postmenopausal women, decline of ovarian function and lack of estrogen disrupt the balance of bone formation and bone absorption during the bone metabolism, leading to an imbalance of the bone phosphorus metabolism and thus ultimately causing osteoporosis. We are interested in whether the bone metabolism is associated with the occurrence of vertebral fractures in osteoporotic patients. Accordingly, the plasma levels of bone metabolic markers were detected in osteoporotic patients with or without vertebral fractures by the ECLIA method. As given in Table 2, the plasma levels of PINP and *β*-CTx were elevated, but the plasma level of 25-(OH)VitD was declined in osteoporotic patients with vertebral fractures when comparable to those without (*P* < 0.05).

### 3.4. The Expression Levels of miR-491-5p and miR-485-3p in Osteoporotic Patients with or without Vertebral Fractures

**Figure 2:**
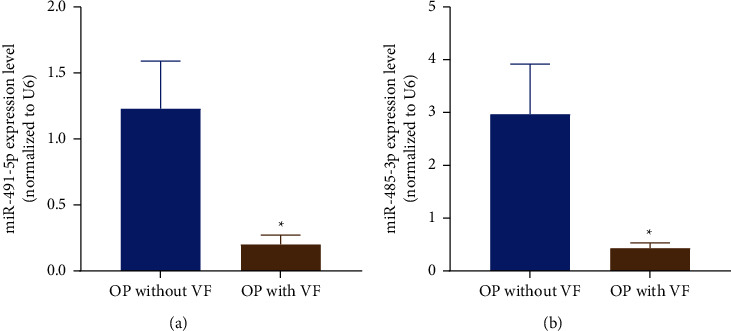
The expression levels of miR-491-5p and miR-485-3p in plasma of osteoporotic patients with or without vertebral fractures. *∗P* < 0.001 compared with osteoporotic patients without vertebral fractures.

To characterize roles of miRNAs in osteoporosis and subsequent incidence risk of vertebral fractures for postmenopausal women, we chose two differentially expressed miRNAs between osteoporotic patients with vertebral fractures and osteoporotic patients without fracture, miR-491-5p and miR-485-3p, and determined their expression levels in plasma of 78 osteoporotic patients with vertebral fractures and 82 osteoporotic patients without vertebral fractures. As shown in Figure 2, the plasma expression levels of miR-491-5p and miR-485-3p were declined in osteoporotic patients with vertebral fractures when comparable to those without (*P* < 0.001), suggesting that reduced expressions of miR-491-5p and miR-485-3p may be involved in the occurrence of vertebral fractures in osteoporotic patients.

### 3.5. Relationship between miR-491-5p and miR-485-3p Expressions and Bone Metabolic Markers

**Figure 3:**
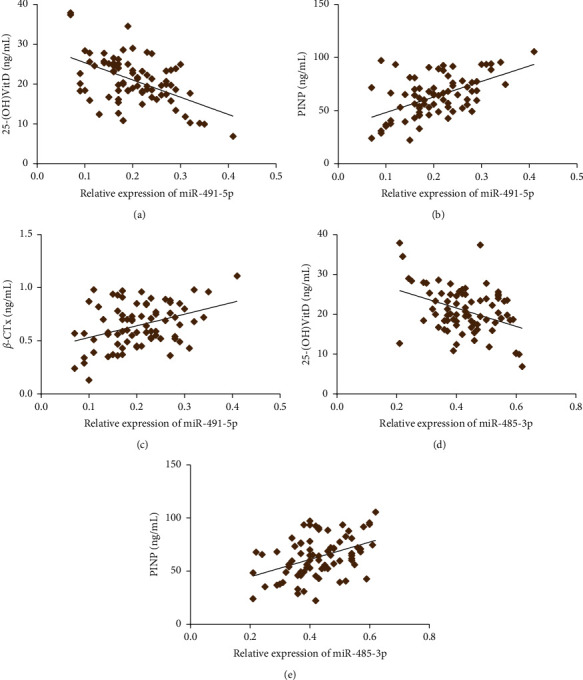
Pearson correlation analysis between miR-491-5p and miR-485-3p expressions, 25-(OH)VitD, PINP, and *β*-CTx.

Pearson correlation analysis (Figure 3) revealed that the relative expression level of miR-491-5p was negatively correlated with the level of 25-(OH)VitD (*r* = −0.518, *P* < 0.001) but positively correlated with the levels of PINP (*r* = 0.547, *P* < 0.001) and *β*-CTx (*r* = 0.380, *P* < 0.001). We also observed a negative correlation between the relative expression level of miR-485-3p and 25-(OH)VitD (*r* = −0.388, *P* < 0.001) and a positive correlation between miR-485-3p and PINP (*r* = 0.422, *P* < 0.001). There was no significant correlation between the relative expression level of miR-485-3p and *β*-CTx (*r* = 0.116, *P* = 0.311).

### 3.6. Predictive Performance of miR-491-5p and miR-485-3p Expressions for the Occurrence of Vertebral Fractures in Osteoporotic Patients

**Figure 4:**
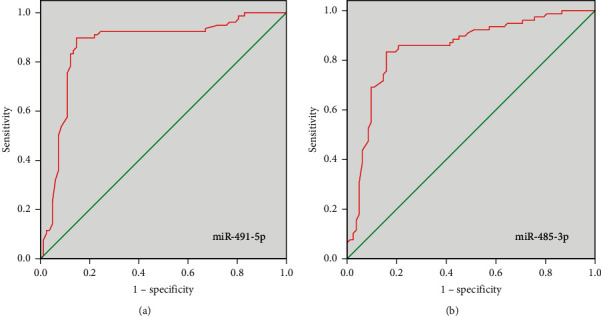
Predictive performance of miR-491-5p and miR-485-3p expressions for the occurrence of vertebral fractures in osteoporotic patients.

ROC curves (Figure 4) for prediction of vertebral fracture following osteoporosis in postmenopausal women by miR-491-5p expression yielded 0.866 AUC with 95% CI 0.803–0.929 (*P* < 0.001) and by miR-485-3p expression produced 0.848 AUC with 95% CI 0.785–0.911 (*P* < 0.001).

## 4. Discussion

The skeletal system plays a major role in supporting body structure and movement and maintaining mineral balance in the human body. Abnormality of the skeletal system will cause a variety of skeletal diseases such as osteoporosis, osteoarthritis, metabolic bone dysplasia, and bone cancer [17]. Osteoporosis is characterized by fragile bones, reduced BMD, and degradation of bone microstructure, leading to an increased risk of vertebral fracture, hip fracture, and fracture in other skeletal sites [18]. The postmenopausal women with osteoporosis are prone to experience vertebral fracture [19]. Candidate biomarkers of osteoporosis may be identified early in the disease, allowing the application of early interventions to prevent further progress of disease. Vitamin D level is negatively associated with age, and vitamin D deficiency is commonly seen in elderly population. Enormous studies have demonstrated that vitamin D deficiency contributed to bone mineralization and bone loss, resulting in osteoporosis and fractures probably [20]. Lower serum (25-(OH)VitD) concentrations were revealed in white women with incident hip fracture than in control participants [21]. However, the black women with higher (25-(OH)VitD) level (≥20 ng/mL) were at higher risk of fracture compared with those with less than 20 ng/mL [22]. The present study investigated that declined plasma 25-(OH)VitD level was shown in osteoporotic patients with vertebral fractures compared with that without fractures. These data suggested that the correlation between vitamin D level and skeletal health might differ by ethnicity. PINP composes three noncovalently linked subunit chains. PINP is a useful biochemical bone turnover marker in bone metastases of osteoblastic nature and bone diseases [23, 24]. Comparing with patients without fractures, the osteoporotic patients with vertebral fractures showed elevated level of PINP in this study. The findings were similar to other study indicating PINP was significantly higher in patients with alcoholic cirrhosis than that in nonalcoholic cirrhosis [25]. Furthermore, the study also found that *β*-CTx level was increased in osteoporotic patients with vertebral fractures than that without fractures. *β*-CTx is a reference bone turnover marker in osteoporosis, and its increase predicts the development of bone diseases [26].

In recent years, the function of gene expression mediated by microRNAs (miRNAs) in pathologic biological processes has been identified. Some circulating miRNAs are involved in the regulation of bone formation and osteocyte differentiation. The previous study revealed miR-23b-3p and miR-140-3p might act as potential biomarkers candidates for osteoporosis in postmenopausal women [27]. The present study identified differentially expressed miRNAs in plasma of osteoporotic patients with and without vertebral fractures based on the GSE93883 dataset, of which miR-491-5p and miR-485-3p expression was analyzed. miR-491-5p plays a role as a tumor suppressor in various cancers. Sun et al. pointed miR-491-5p is downregulated in gastric cancer tissues compared with adjacent noncancerous tissues. miR-491-5p mediated by Foxi1 suppressed gastric cancer progression through inhibiting Wnt3*α*/*β*-catenin signaling [28]. Chen et al. explored miR-491-5p level declined in osteosarcoma and miR-491-5p inhibited osteosarcoma cell proliferation by targeting PKM2 [29]. These outcomes were in accordance with our finding, which demonstrated, comparing with the patients without fractures, the osteoporotic patients with vertebral fractures had declined miR-491-5p expression. Furthermore, declined expression of miR-485-3p was revealed in patients with vertebral fractures. miR-485-3p is located in the 101055419-101055491 (+) gene region on chromosome 14q32 where mutations are frequently observed in cancers. The promoting or inhibiting effect of mir-485-3p in cancer has been determined in a large number of studies. Overexpression of miR-485-3p promoted the development of gastric cancer [30], hepatocellular carcinoma [31], and prostate cancer [32]. In contrast, miR-485-3p expression can suppress relevant tumors in osteosarcoma [33], small lung cancer [34], and glioblastoma [35]. These contrasting findings may be related to different tumor tissue structures and disease pathogeneses. Pearson correlation analysis in this study indicated that relative expression levels of miR-491-5p and miR-485-3p were positively correlated with the levels of PINP but negatively correlated with 25-(OH)VitD level, and miR-491-5p expression was positively correlated with *β*-CTx. ROC curves revealed miR-491-5p and miR-485-3p expressions can be as biomarkers to predict vertebral fractures in osteoporotic patients.

This retrospective study analyzed the function of miRNAs in osteoporotic postmenopausal women with vertebral fractures and investigated the expression of miR-491-5p and miR-485-3p. All data revealed miR-491-5p and miR-485-3p may be involved in the occurrence of vertebral fractures in osteoporotic patients and predict the incidence risk of vertebral fractures for postmenopausal women. However, insufficient samples may affect the reliability of the data in this study. Further investigations were warranted to identify target genes of miR-491-5p and miR-485-3p to affect vertebral fracture following osteoporosis considering the fact that miRNAs posttranscriptionally modulate and silence target gene expression.

## Figures and Tables

**Table 1:** Differentially expressed miRNAs that were upregulated or downregulated |log2 (fold change)|  ≥ 2 (corrected *P* < 0.05) between osteoporotic patients with vertebral fractures and osteoporotic patients without fracture.

miRNA_ID	Log2 (fold change)	Adj. *p* value
hsa-miR-31-5p	−5.041223	0.008438
hsa-miR-30c-2-3p	−2.196561	0.030113
hsa-miR-432-5p	−2.731635	0.035109
hsa-miR-3920	2.726748	0.00341
hsa-miR-491-5p	3.32026	0.004932
hsa-miR-576-5p	2.287523	0.004932
hsa-miR-647	2.260594	0.01109
hsa-miR-4768-5p	2.299533	0.01417
hsa-miR-3183	2.18766	0.01417
hsa-miR-4756-5p	2.220542	0.01417
hsa-miR-4756-3p	2.442396	0.015896
hsa-miR-2114-5p	2.042855	0.019263
hsa-miR-4305	2.276466	0.025967
hsa-miR-3119	2.471177	0.032647
hsa-miR-676-5p	2.061763	0.032647
hsa-miR-485-3p	3.032694	0.038006
hsa-miR-4268	2.465008	0.048771

**Table 2:** The plasma levels of 25-(OH)VitD, PINP, and *β*-CTx in osteoporotic patients with or without vertebral fractures.

Group	25-(OH)VitD (ng/mL)	PINP (ng/mL)	*β*-CTx (ng/mL)
Osteoporotic patients with vertebral fractures (*n* = 78)	20.89 ± 5.87	63.37 ± 19.16	0.64 ± 0.20
Osteoporotic patients without vertebral fractures (*n* = 82)	25.83 ± 6.89	43.27 ± 15.73	0.46 ± 0.17
*t*	4.870	7.268	6.144
*P*	<0.001	<0.001	<0.001

## Data Availability

The data used to support the findings of this study are included within the article.
